# Escaping Death: Mitochondrial Redox Homeostasis in Cancer Cells

**DOI:** 10.3389/fonc.2017.00117

**Published:** 2017-06-09

**Authors:** Francesco Ciccarese, Vincenzo Ciminale

**Affiliations:** ^1^Department of Surgery, Oncology and Gastroenterology, University of Padua, Padua, Italy; ^2^Veneto Institute of Oncology – IRCCS, Padua, Italy

**Keywords:** mitochondria, reactive oxygen species, cancer metabolism, nicotinamide adenine dinucleotide phosphate, apoptosis

## Abstract

Reactive oxygen species (ROS) are important signaling molecules that act through the oxidation of nucleic acids, proteins, and lipids. Several hallmarks of cancer, including uncontrolled proliferation, angiogenesis, and genomic instability, are promoted by the increased ROS levels commonly found in tumor cells. To counteract excessive ROS accumulation, oxidative stress, and death, cancer cells tightly regulate ROS levels by enhancing scavenging enzymes, which are dependent on the reducing cofactor nicotinamide adenine dinucleotide phosphate (NADPH). This review focuses on mitochondrial ROS homeostasis with a description of six pathways of NADPH production in mitochondria and a discussion of the possible strategies of pharmacological intervention to selectively eliminate cancer cells by increasing their ROS levels.

## Introduction

Almost all eukaryotic cells rely on molecular oxygen (O_2_) to derive energy from nutrients. The mitochondrial electron transport chain (ETC) couples the transfer of electrons to O_2_ with active proton pumping from the mitochondrial matrix to the intermembrane space (IMS). The resulting proton motive force is used to drive production of ATP by the F_0_F_1_ mitochondrial ATP synthase complex in the process known as oxidative phosphorylation.

Incomplete reduction of O_2_ gives rise to reactive oxygen species (ROS). One-electron reduction of O_2_ to superoxide (${\text{O}}_{2}^{-•}$) can occur in 11 different sites within mitochondria (1, 2). Most of ${\text{O}}_{2}^{-•}$ is produced as a consequence of an electron leak from ETC complexes I, II, and III. The latter is the only complex able to generate ${\text{O}}_{2}^{-•}$ both in the matrix and IMS, while complexes I and II produce ${\text{O}}_{2}^{-•}$ exclusively in the matrix (3, 4). ${\text{O}}_{2}^{-•}$ generated in the matrix is rapidly converted to hydrogen peroxide (H_2_O_2_) by manganese superoxide dismutase (MnSOD or SOD2) (5), whereas ${\text{O}}_{2}^{-•}$ in the IMS diffuses through the voltage-dependent anion channels (6) into the cytosol, where it is converted to H_2_O_2_ by Cu/Zn superoxide dismutase (SOD1).

## ROS as Signaling Molecules in Normal and Cancer Cells

Long considered as harmful by-products of oxidative metabolism, ROS have recently been recognized as important signaling molecules (7, 8). Given its higher intracellular concentration and permeability through biological membranes, H_2_O_2_ is considered the principal ROS involved in signaling, mainly through its ability to induce reversible cysteine oxidation in redox-sensitive proteins. Cysteine oxidation to sulfenic acid (SO^−^) is estimated to occur in the presence of nanomolar concentrations of H_2_O_2_ (9); this modification profoundly affects the activity of several target proteins. One well-characterized example is the oxidation of cysteine residues in the phosphatase PTEN, leading to its inactivation and enhanced PI3K-AKT signaling (10). Sustained oxidative inhibition of protein phosphatases DEP-1/PTPRJ, PTP1B, and SHP2 (11, 12) also results in the deregulation of mitogenic and survival pathways, such as MAPK-ERK and AKT. On the other hand, cysteine oxidation of the epidermal growth factor receptor promotes its activity (13). Mitochondrial ROS (mtROS) also play an important role in T cell activation. In fact, T cell receptor (TCR) signaling leads to Ca^2+^ release from the endoplasmic reticulum and uptake by mitochondria, where it stimulates the activity of the tricarboxylic acid (TCA) cycle. As a consequence, mitochondrial respiration is promoted through increased NADH and succinate production, which fuel the ETC, leading ultimately to increased mtROS generation. H_2_O_2_ generated in the cytosol from complex III-derived ${\text{O}}_{2}^{-•}$ enhances the nuclear localization of NFAT, a transcription factor that drives the expression of *IL2* and other genes related to T cell activation (14). Moreover, following TCR activation, excess glycerol-3-phosphate is produced by glycolysis and taken up by mitochondria, where it is oxidized by glycerol-3-phosphate dehydrogenase. This reaction increases electron flow through the coenzyme Q pool and electron leak from ETC complexes (15). Reactive oxygen species modulator 1, a redox sensor frequently overexpressed in human tumors, is also capable of increasing mtROS (16) and controls the G1-S checkpoint of the cell cycle by regulating the expression of p27^Kip1^ (17). Another important ROS-producing protein is p66Shc, which has been implied in oxidative stress and apoptosis induction (18). In mitochondria, p66Shc is activated through phosphorylation of a serine residue by the ROS-sensitive protein kinase Cβ (19). Once activated, p66Shc increases ROS levels through different mechanisms. As an adaptor protein, p66Shc recruits the nucleotide exchange factor SOS, leading to the activation of small GTPase Rac-1, which ultimately promotes the assembly of membrane-associated NADPH-oxidases and ROS production (20). Moreover, it has been suggested that p66Shc may directly transfer electrons from cytochrome *c* to O_2_ (21), thus increasing mtROS production. ROS-induced ROS production promoted by the activation of p66Shc is a signaling mechanism involved in apoptosis (22), aging (23), and also growth factor-induced cell migration (11), proliferation of cancer cells (24), and angiogenesis (25). ROS production in mitochondria can also be sustained by outer mitochondrial membrane-bound l-monoamine oxidases (MAO), a family of flavoproteins that catalyze the oxidative deamination of monoamines. Humans produce two types of MAO, named MAO-A and MAO-B, both of which are distributed throughout the body, with particularly high expression in the central nervous system (26); fibroblasts and placenta produce only MAO-A (27), while platelets and lymphocytes express only MAO-B (28). MAO-A and MAO-B are involved in the inactivation of neurotransmitters. Serotonin, melatonin, epinephrine, and norepinephrine are metabolized by MAO-A, and phenethylamine and benzylamine are metabolized by MAO-B, while both forms metabolize dopamine, tyramine, and tryptamine (29). The conversion of monoamines to aldehydes and ammonia requires O_2_ and is associated with H_2_O_2_ production (30, 31). MAO-A activation in monocytes/macrophages has been demonstrated to induce an anti-inflammatory state through H_2_O_2_-mediated inhibition of inducible nitric oxide synthase (32). mtROS production through MAO is also involved in pathological states, such as ischemia–reperfusion (33), heart failure (34), neurodegeneration (35), and vascular dysfunction (36). Electron leakage from the ETC and NADPH oxidase 4 activity accounts for mitochondrial ${\text{O}}_{2}^{-•}$ production, which is rapidly dismutated to H_2_O_2_. In addition, MAO-A and MAO-B contribute to increase H_2_O_2_ load inside mitochondria.

Given the important roles of ROS in mitogenic signaling, it is not surprising that cancer cells produce large amounts of H_2_O_2_ (37) and that aberrant activation of oncogenes and/or loss of tumor suppressor genes increase ROS production in cancer. Notably, important aspects of cancer cell biology appear to be mediated by increased mtROS production driven by oncogenic signaling. Mutations activating KRAS are associated with increased production of ROS from the Q_o_ site of complex III (38); the resulting increase in mtROS generation dampens excessive ERK1/2 activation that would otherwise result in growth arrest. c-Myc, another important oncogene frequently activated in cancer, induces mitochondrial biogenesis, thus affecting mtROS production (39). Interestingly, mtROS were also shown to enhance the stabilization of hypoxia-inducible factor-1α (HIF-1α) (40), which engages key oncogenic pathways, resulting in alterations of the metabolic profile, neoangiogenesis, and metastasis (41). Increased mtROS production may also result in a vicious circle by inducing mitochondrial DNA mutations that may impair ETC function, further enhancing electron leak and ROS generation.

The proliferative/survival advantage contributed by increased ROS levels is not devoid of “side effects,” as sustained accumulation of H_2_O_2_ in the presence of Fe^2+^ or Cu^2+^ leads to the formation of the highly damaging hydroxyl radical (OH^•^). Moreover, when the cytosolic concentration of H_2_O_2_ exceeds the nanomolar range, SO^−^ is further oxidized to sulfinic $\left({\text{SO}}_{\text{2}}^{-}\right)$ and then to sulfonic $\left({\text{SO}}_{\text{3}}^{-}\right)$ acid, leading to irreversible protein modification (9). Consequent inactivation of proteins essential for cell survival, along with widespread macromolecular damage, results in cell death. Given their role in both mitogenic signaling and apoptosis, ROS are viewed as either “friends or foes” in the cancer transformation process. On one hand, sustained ROS production may be advantageous to cancer cells through the increased activation of mitogenic signaling pathways (42), on the other ROS enhance the sensitivity of cancer cells to apoptosis and may induce further ROS production (43–47). Experimental evidence suggests that cell fate—growth arrest, proliferation, or death—is decided by a ROS rheostat, whose levels are associated with different biological responses (48). While in normal cells, the ROS rheostat is usually set to a low level, in cancer cells the rheostat is set to an intermediate level necessary to sustain tumorigenicity. Further increases of ROS levels lead to oxidative macromolecular damage and selective elimination of cancer cells. Thus, modulation of ROS levels is a promising anticancer strategy (45). As mitochondria are pivotal for life and death decisions and several mitochondrial proteins are ROS-sensitive, including aconitase, isocitrate dehydrogenase (IDH), succinate dehydrogenase, complex I of the ETC, and F_0_F_1_ mitochondrial ATP synthase (49)—mtROS could play a very important role in determining cell fate decisions. However, mtROS production is tightly regulated by O_2_ availability, by the concentration and redox state of the electron carriers NADH and FADH_2_, and by mitochondrial membrane potential (50, 51). Scavenging of mtROS is also tightly controlled by mitochondrial isoforms of key enzymes (e.g., superoxide dismutase 2, glutathione peroxidase, thioredoxin 2, and thioredoxin reductase 2) and by the mitochondrial nicotinamide adenine dinucleotide phosphate (NADPH) pool.

## NADPH: The Universal Source of Reducing Equivalents Utilized by ROS-Scavenging Systems

In addition to ROS-generating pathways, antioxidant/scavenger systems play a key role in maintaining ROS homeostasis. H_2_O_2_ is kept below toxic levels by two principal enzymatic systems that reduce H_2_O_2_ to H_2_O: thioredoxin/peroxiredoxin (TRX/PRX) and glutathione/glutathione peroxidase (GSH/GPX). The two principal cellular antioxidants (GSH and TRX) act as electron donors to reduce oxidized macromolecules, becoming themselves oxidized in the process. Oxidized glutathione (GSSG) and TRX may then be restored in their reduced state through the action of glutathione reductase and thioredoxin reductase, respectively, using NADPH as the electron donor. This process generates NADP^+^, which may be reconverted to NADPH using electrons obtained from different oxidative biochemical pathways.

The oxidative branch of the pentose phosphate pathway (PPP) is commonly considered the principal contributor to the NADPH pool in the cytosol (52). The first two enzymes of this pathway, glucose-6-phosphate dehydrogenase (G6PD) and 6-phosphogluconate dehydrogenase each generate an NADPH molecule and produce intermediates used for nucleotide synthesis. In order to cope with oxidative stress and to increase nucleotide synthesis, cancer cells enhance glucose flux through the PPP [for a recent review, see Ref. (53)]. Glucose catabolism thus provides the cytosol with the reducing potential required to maintain redox homeostasis and sustain anabolic processes. It is noteworthy that the pyridine nucleotide pools are highly compartmentalized inside the cell, and no intracellular transport systems for NADPH or NADH have been described so far (54). As mentioned above, mitochondria are the principal site of ROS generation; however, the PPP is only present in the cytosol, so other biochemical pathways must be operative in mitochondria in order to preserve the NADPH pool.

Mitochondria are known to play a central role in the intrinsic apoptosis pathway by sequestering key proapoptotic factors, the release of which can be promoted by oxidative activation of permeability transition pore (55, 56) or through ROS-mediated opening of the BAX/BAK “gateway” (57). Consistent with this notion, mitochondrial redox homeostasis is particularly critical in cell viability.

Six different NADPH-producing pathways are present in mitochondria: (i) NADP^+^ transhydrogenation by nicotinamide nucleotide transhydrogenase (NNT) using NADH as a cofactor; (ii) glutamate conversion to α-ketoglutarate by glutamate dehydrogenase 1 (GDH1); (iii) NADH phosphorylation by mitochondrial NAD kinase (NADK2); (iv) isocitrate dehydrogenase 2 (IDH2); (v) malic enzymes (ME2/3); and (vi) the mitochondrial folate cycle. Notably, the latter pathway has recently emerged as the principal contributor to the maintenance of the NADPH pool in cancer cells (58). Scavenging of mitochondrial H_2_O_2_ is particularly critical for cancer cell viability, as mtROS induce cytochrome *c* release from mitochondria thus enhancing sensitivity to apoptotic stimuli (59). Pharmacological targeting of NADPH-producing pathways in mitochondria might therefore be an interesting means to prime cancer cells to apoptosis.

## Nicotinamide Nucleotide Transhydrogenase

**Figure 1:**
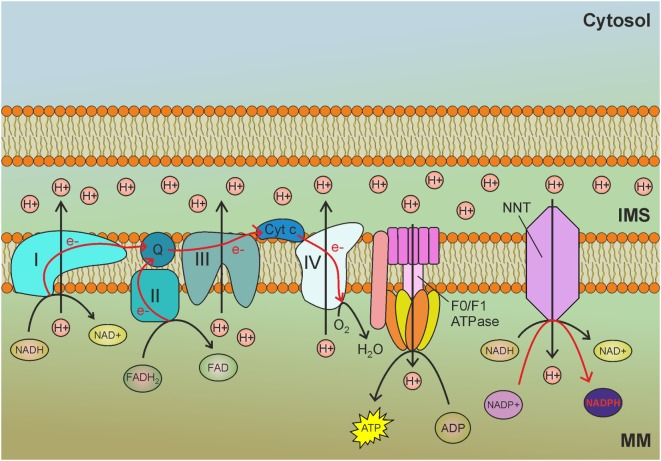
Nicotinamide nucleotide transhydrogenase (NNT) exploits the mitochondrial membrane potential generated by the electron transport chain (ETC) to reduce NADP^+^ to nicotinamide adenine dinucleotide phosphate (NADPH) by transhydrogenation with NADH. MM, mitochondrial matrix; IMS, intermembrane space; I, complex I of the ETC; II, complex II of the ETC; III, complex III of the ETC; IV, complex IV of the ETC; Q, ubiquinone; Cyt *c*, cytochrome *c*; e−, electrons. Red arrows indicate reactions involved in NADPH production.

**Figure 2:**
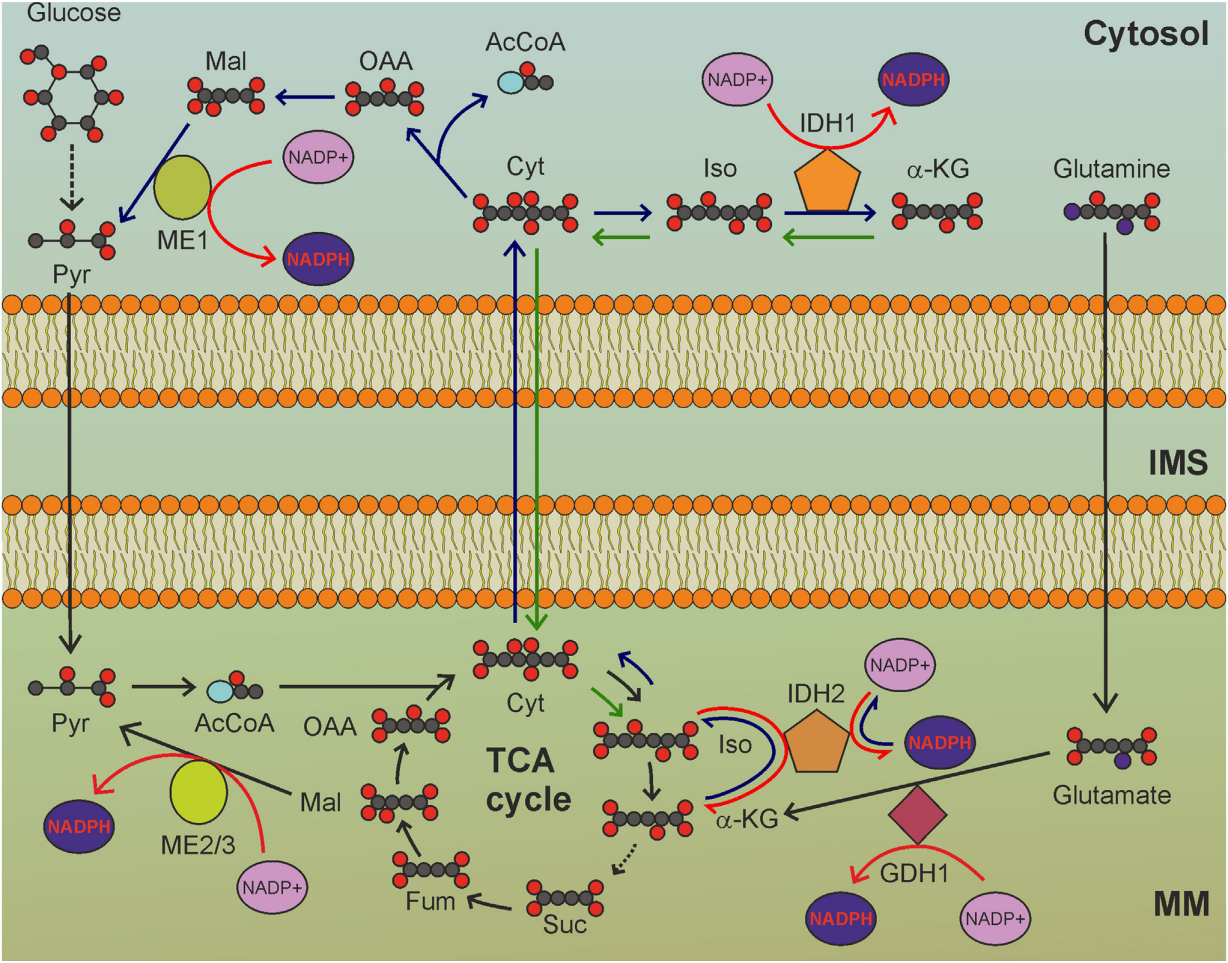
The tricarboxylic acid (TCA) cycle is a hub for mitochondrial nicotinamide adenine dinucleotide phosphate (NADPH) production. GDH1-, IDH2-, and ME2/3-catalyzed reactions involve intermediates of the TCA cycle to reduce NADP^+^ to NADPH. MM, mitochondrial matrix; IMS, intermembrane space; Pyr, pyruvate; AcCoA, acetyl-CoA; Cit: citrate; Iso, isocitrate; α-KG, α-ketoglutarate; Suc, succinate; Fum, fumarate; Mal, malate; OAA, oxaloacetate. Gray circles indicate carbon atoms, red circles indicate oxygen atoms, blue circles indicate nitrogen atoms, and yellow circles indicate phosphate groups. Hydrogen atoms are omitted. Light blue oval: coenzyme A. Dark blue arrows indicate reactions involved in reductive carboxylation, green arrows indicate reactions involved in reverse reductive carboxylation. Red arrows indicate reactions involved in NADPH production. NADH and FADH_2_ production by TCA cycle, as well as release of CO_2_ by oxidative decarboxylation of isocitrate, are not shown.

Nicotinamide nucleotide transhydrogenase (NNT) is an integral protein of the inner mitochondrial membrane that uses the proton motive force generated by the ETC to catalyze the transfer of hydride (H^−^) from NADH to NADP^+^ (Figure 1). About half of the mitochondrial NADPH pool is sensitive to uncouplers (60) and is thus likely to be generated by NNT. However, NNT is likely to be inhibited in conditions of high NADPH/NADP^+^ ratio (61) and would be activated in conditions that lead to NADPH consumption. In addition to maintaining elevated NADPH/NADP^+^ and GSH/GSSG ratios in mitochondria, NNT plays a key role in coordinating glucose catabolism and glutaminolysis. The latter replenishes intermediates of the TCA cycle (anaplerotic pathway) through three sequential reactions: (i) glutaminase-catalyzed hydrolysis of glutamine to glutamate, (ii) conversion of glutamate to α-ketoglutarate by glutamate dehydrogenase (GDH) reaction that generates NADPH, and (iii) reductive carboxylation of α-ketoglutarate to isocitrate, a reaction that requires NADPH and is thus sustained by NNT and other pathways leading to mitochondrial NADPH production (Figure 2) (62). Consistent with this notion, NNT knockdown in melanoma cells reduces both NADPH production in mitochondria and glutamine catabolism while increasing flow of glycolytically derived pyruvate into the TCA cycle through the activation of pyruvate carboxylase to maintain TCA anaplerosis (62). Notably, glutaminolysis, increased glucose uptake, and glycolysis are metabolic adaptations necessary to support increased proliferation and, thus, are characteristic of most cancer cells. Hence, NNT appears to play a central role in the metabolic reprogramming of cancer cells. NADPH obtained by transhydrogenation is also used for the reductive carboxylation of α-ketoglutarate to isocitrate by IDH2. Isocitrate can then be converted to citrate, which may be exported to the cytosol and used for lipogenesis (61).

The role of NNT-derived NADPH in ROS scavenging has been assessed in *Caenorhabditis elegans* (63), in which deletion of *nnt-1* results in a fivefold drop in the GSH/GSSG ratio and increased sensitivity to Paraquat-induced oxidative stress compared to wild-type worms. Despite the decreased respiration observed in some tumors, cancer cells often exhibit hyperpolarized mitochondria (64), which stimulate the activity of NNT. Moreover, through the malate-aspartate shuttle, the NADH generated in the cytosol by increased glycolysis can be transferred to mitochondria where it drives NADP^+^ transhydrogenation. Overall, NNT activity plays a very important role in tumorigenesis, both by sustaining reductive carboxylation and by maintaining a high GSH/GSSG ratio during oxidative stress; this may be particularly relevant in hypoxic areas of the tumor, where mtROS production is increased and the concurrent elevated NADH/NAD^+^ ratio may sustain NNT activity.

NNT silencing has been demonstrated to reduce mitochondrial NADPH levels and to increase sensitivity to oxidative stress (65). In fact, a strategy leading to the blockade of anabolic pathways and mitochondrial oxidative stress would be very promising for cancer treatment. Unfortunately, pharmacological inhibition of NNT *in vivo* is not currently feasible, as NNT-specific drugs have not been developed yet. As in the case of the ATP synthase and many metabolic enzymes, NNT activity can be reversed. The transhydrogenation of NAD^+^ by NADPH oxidation is a thermodynamically unfavorable process due to the elevated NADH/NAD^+^ ratio in mitochondria. However, cancer cells frequently overexpress UCP2, which is activated by ${\text{O}}_{2}^{-•}$, and dissipates the proton motive force (66). This could enhance NADH oxidation at the level of complex I, reducing the NADH/NAD^+^ ratio. In combination with a strategy aimed at decreasing NADH production, such as glycolysis inhibition, NADPH-NAD^+^ transhydrogenation could be favored to maintain the NADH pool. Reversed NNT activity has been observed in the failing heart, where increased NAD^+^ levels in fact drive NADPH-NAD^+^ transhydrogenation (67). The possibility to use NNT reversion as a strategy to selectively kill cancer cells needs further investigation.

## Glutamate Dehydrogenase 1

Glutamate dehydrogenase (GDH) generates NADPH or NADH through the conversion of glutamate to α-ketoglutarate (Figure 2). The relative affinities of GDH1 for NAD^+^ and NADP^+^ were not evaluated in humans. Experiments carried out *in vitro* using GDH1 purified from bovine brain indicated that the dissociation constants were 0.83 mM for NAD^+^ and 1.22 mM for NADP^+^ (68). Two GDH isoforms exist in humans: GDH1, which is ubiquitously expressed, and the neuronal GDH2, originated by retrotransposition of the *GLUD1* gene (encoding GDH1) to the X chromosome. GDH1 is the second enzyme of the glutaminolytic pathway. Breast cancer and lung cancer cells overexpress GDH1 (69). Interestingly, GDH1 activity produces different effects depending on the metabolic profile of cancer cells and the prevailing fate of the α-ketoglutarate produced by GDH1. In cancer cells with prevailing reductive carboxylation of α-ketoglutarate by IDH2, GDH1-derived NADPH is consumed to support this reaction. On the contrary, in cancer cells with prevailing oxidative decarboxylation of α-ketoglutarate (i.e., forward TCA cycle), a reaction that does not consume NADPH, GDH1 overexpression leads to increased mitochondrial production of NADPH. In cells with forward TCA cycle, the increased fumarate levels, which result from α-ketoglutarate production by GDH1, activate glutathione peroxidase 1 (GPx1) by directly binding to the enzyme (69), thus linking GDH1 to increased ROS scavenging. GDH1 knockdown impairs proliferation of breast and lung cancer cells, as well as erythroleukemia cells, while it does not affect proliferation of normal cells, which are not dependent on GDH1 to derive mitochondrial NADPH. Moreover, GDH1-deficient cancer cells have increased mtROS levels and decreased NADPH and GSH levels, which would increase oxidative stress and may result in cytotoxicity.

One important metabolic characteristic of proliferating cells, including cancer cells, is increased demand for nitrogen (70), necessary for the synthesis of numerous metabolites, including amino acids. Cancer cells meet an elevated demand for amino acid production through TCA cycle cataplerosis of α-keto acids, which are subsequently transaminated with glutamate to obtain the different non-essential amino acids. GDH1 knockdown blunts the fueling of glutamine into the TCA cycle, thus transiently depleting the α-keto acids necessary for amino acid synthesis. α-ketoglutarate is the universal α-keto acid used in the transamination reactions that are required in amino acid catabolism. To avoid a lethal blockade in nitrogen flow, cancer cells compensate the depletion of α-keto acids by increasing the contribution of glucose to TCA cycle anaplerosis, thus becoming glucose-addicted (69). An important consequence of these changes could be the diversion of glucose flow from the PPP, leading to a decrease in NADPH production in the cytosol. Surprisingly, no difference in oxidative PPP (oxPPP) flow was observed in GDH1-depleted cells compared to control cells (69). However, these conclusion was based on experiments using d-[U-^14^C]glucose labeling, which does not distinguish the metabolic source of ^14^CO_2_. In fact, using uniformly labeled glucose, ^14^CO_2_ is released from the oxidative decarboxylation reactions involving pyruvate, isocitrate, α-ketoglutarate, and 6-phosphogluconate. GDH1 knockdown therefore produces complex metabolic effects in cancer cells, leading to increased sensitivity to glucose deprivation and altered redox balance, probably in part through impairment of oxPPP. As GDH1 knockdown drives TCA cycle dependence on glucose, GDH1 activity would be expected to be necessary to promote cancer survival under glucose deprivation. Studies carried out in glioblastoma cells suggest that this is in fact the case (71). Normal cells respond to glucose deprivation by activating β-oxidation of fatty acids, while cancer cells with c-Myc amplification avoid depletion of TCA cycle intermediates by increasing glutamine utilization through GDH1. In glioblastoma cells, loss of glucose is not compensated by the utilization of amino acids, but increases the generation of ammonia. This indicates that when glucose is limiting, glutamine is channeled in catabolic reactions at the expense of other glutamate-based reactions (i.e., transamination reactions). The ratio of GDH to alanine aminotransferase activity is increased 12-fold in glucose-deprived glioblastoma cells (71). Overall, GDH1 activity in cancer cells seems to be more related to metabolic adaptation to nutrient starvation than to redox balance maintenance.

However, GDH1 is highly expressed in most cancers and its inhibition proved to be efficient in impairing redox homeostasis (69). The green tea-derived polyphenol epigallocatechin gallate (ECGC) inhibits several proteins, including GDH1. More specific and more potent, the purpurin analog R162 is a very promising drug that decreases fumarate levels, thus diminishing GPx1 activity and increasing ROS levels in lung- and breast-cancer cells, without affecting proliferation of normal cells. Moreover, R162 also significantly slows down tumor growth *in vivo*, suggesting GDH1 as an attractive target in anticancer therapies (69).

## Mitochondrial NAD Kinase

**Figure 3:**
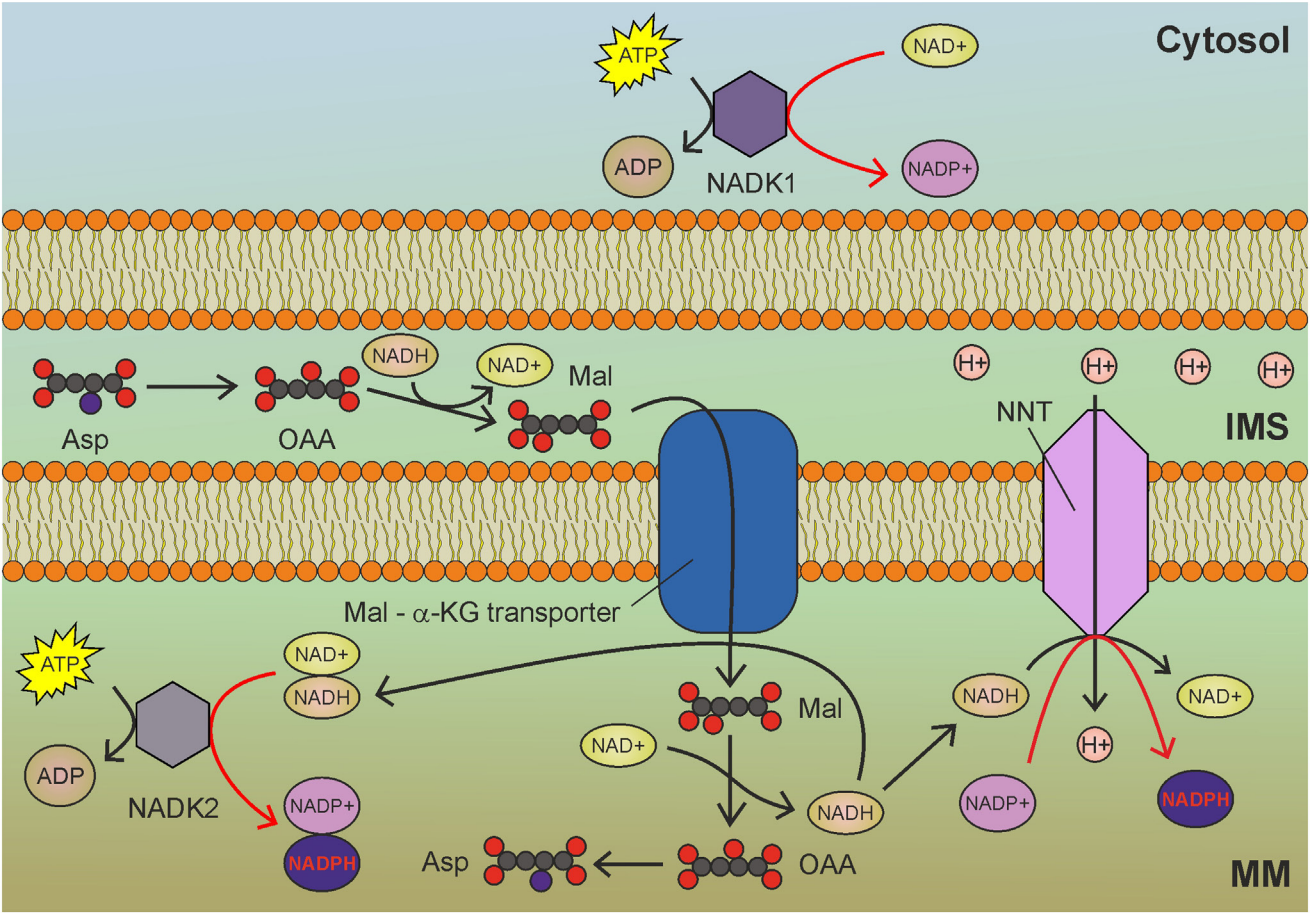
Mitochondrial NAD kinase (NADK2) uses ATP to phosphorylate NADH to obtain nicotinamide adenine dinucleotide phosphate (NADPH) in mitochondria. NADH derived by glycolysis and tricarboxylic acid cycle drive NADPH production through NADK2 and NNT. MM, mitochondrial matrix; IMS, intermembrane space; Asp, aspartate; OAA, oxaloacetate; Mal, malate; α-KG, α-ketoglutarate. Gray circles indicate carbon atoms, red circles indicate oxygen atoms, and blue circles indicate nitrogen atoms. Hydrogen atoms are omitted. Red arrows indicate reactions involved in NADPH production.

NAD^+^ kinase (NADK) phosphorylates NAD^+^ at the 2′ position of the ribose ring using ATP as a phosphate donor, thus producing NADP^+^ (Figure 3). NADK is solely responsible for NADP biosynthesis by the phosphorylation of NAD. As the redox function of NADP^+^ results from its interconversion between oxidized and reduced forms, the necessity for *de novo* NADP^+^ synthesis through NAD^+^ phosphorylation is not obvious. However, NADK knockdown leads to about 70% loss of the overall NADPH pool and to increased sensitivity to oxidative stress (72). Why is *de novo* synthesis of NADP^+^ so important? Besides being redox regulators, both NAD^+^ and NADP^+^ are precursors of important signaling molecules. Two-second messengers are derived from NADP^+^: the phosphorylated form of cyclic ADP-ribose and nicotinic acid adenine dinucleotide phosphate (NAADP), both of which are generated by ADP-ribosyl cyclases and are involved in Ca^2+^ release from intracellular stores (73). The NADP^+^ pool is needed to sustain calcium signaling, thus rendering NADP biosynthesis crucial for cell viability. NADP^+^ pool depletion could be particularly relevant in cancer cells, which use NAADP-dependent calcium signaling to sustain tumor progression, metastasis, and angiogenesis (74, 75). The NAD^+^ used as a precursor in NADP^+^ synthesis can in turn be synthesized *de novo* from l-tryptophan and nicotinic acid.

The best characterized isoform of NADK in mammalian cells is the cytosolic NADK1 (Figure 3), identified in 2001 (76), which is involved in the maintenance of the cytosolic NADP pool. Given the compartmentalized nature of NAD and NADP, the existence of a mitochondrial isoform of NADK was postulated. However, NADK1 remained the unique human NADK for a decade, until the identification of NADK2 (also known as MNADK) (77, 78), which is responsible for the maintenance of the mitochondrial NADPH pool. An interesting feature of mitochondrial NADK (Figure 3) is that, notwithstanding its preference for NAD^+^, it can also phosphorylate NADH, directly generating NADPH (77). This latter activity could rapidly generate mitochondrial NADPH (at the expense of ATP) to counteract oxidative stress. As the malate-aspartate shuttle transfers NADH from the cytosol to mitochondria, increased glycolysis could contribute to mitochondrial redox balance by sustaining the activity of both NNT and NADK2 (Figure 3).

NAD^+^ kinase activity is regulated by the redox state of pyridine nucleotides. NADH, NADPH and elevated concentrations of NADP^+^ inhibit NADK1 and NADK2 (79). Interestingly, NADK activity is promoted by calcium through direct binding to the calcium-binding protein calmodulin (80). This raises the possibility that NADP biosynthesis could be slowed down in mitochondria of cancer cells by reducing calcium levels. NADK activity is inhibited by thionicotinamide adenine dinucleotide and thionicotinamide adenine dinucleotide phosphate, which are derived from NAD and NADP, respectively. These compounds increase oxidative stress and also synergize with chemotherapy *in vivo* (81). Inhibitors of NADK could be very promising anticancer agents, as NADK inhibition leads to degradation of dihydrofolate reductase (DHFR) due to reduced levels of NADPH, which is required for DHFR stability (82). The resulting impairment of the folate cycle impinges on the *de novo* synthesis of purines, thymidylate, and certain amino acids. Given its recent discovery, little is known about NADK2 and its role in cancer and other pathologies.

## Isocitrate Dehydrogenase 2

Isocitrate dehydrogenases catalyze the oxidation of isocitrate to oxalosuccinate and then to α-ketoglutarate, with the release of CO_2_ (Figure 2). In mitochondria, the oxidative decarboxylation of isocitrate takes place in the context of the TCA cycle and is catalyzed by the NAD-dependent IDH3 isoform. The IDH1 and IDH2 isoforms, instead, are NAD(P)H-dependent enzymes, which can use both NAD^+^ or NADP^+^ and operate outside the TCA cycle in the cytosol and in mitochondria, respectively. While IDH3 is only able to perform oxidative decarboxylation, IDH1 and IDH2 also catalyze the reverse reaction, i.e., reductive carboxylation (83). In its oxidative decarboxylation mode, IDH2 contributes to mitochondrial NADPH pool maintenance (84). Consistent with this notion, lack of IDH2 activity increases mtROS levels and sensitizes cells to oxidative stress (85). Interestingly, forward IDH2 activity is increased by the NAD^+^-dependent deacetylase SIRT3 (86). NADH oxidation by the ETC and NAD^+^ production by the NNT enhance SIRT3-mediated deacetylation of IDH2 and production of NADPH in mitochondria. In normal cells, mitochondrial respiration supports SIRT3 activation, leading to enhanced oxidative decarboxylation of isocitrate to α-ketoglutarate by IDH2, with concomitant production of NADPH.

In cancer cells, the situation is far more complicated. In fact, monoallelic mutations at Arg172 in *IDH2* frequently found in gliomas, glioblastomas, and acute myeloid leukemia (87) result in a neomorphic function, with the production of D-2-hydroxyglutarate from α-ketoglutarate and consumption of NADPH. In contrast, tumors with wild-type IDH2 produce NADPH through the oxidative decarboxylation of isocitrate to α-ketoglutarate. Consistent with this notion, in melanoma cells, which in most cases do not carry *IDH2* mutations, knockdown of this gene decreases the mitochondrial NADPH/NADP^+^ ratio, increases ROS levels, and impairs tumorigenicity *in vivo* (88). IDH2 knockdown also increases the sensitivity of melanoma cells to the ROS-inducer compound emodin (89).

Although melanoma cells seem to rely on IDH2 activity to maintain proper mitochondrial redox balance, most other types of cancer cells depend on IDH2 for reductive carboxylation. In this process, citrate is exported to the cytosol in order to sustain anabolic processes. To avoid depletion of TCA cycle intermediates, glutamine is converted to glutamate, which is subsequently converted to α-ketoglutarate with NADPH (or preferentially NADH) production. Citrate export from mitochondria to cytosol is sustained by α-ketoglutarate conversion to isocitrate by IDH2 with NADPH consumption (Figure 2). NADH generated by GDH1 activity could be used to produce NADPH in the transhydrogenation reaction. Thus, the NADPH balance of reductive carboxylation in mitochondria is null (Figure 2). In the cytosol, reductive carboxylation increases NADPH levels, as citrate is hydrolyzed to oxaloacetate and acetyl-CoA. Oxoloacetate is, then, converted to malate, which is finally converted to pyruvate by malic enzyme 1 (Figure 2), producing NADPH. Moreover, the NADPH-dependent production of the oncometabolite D-2-hydroxyglutarate by mutant IDH2 inhibits histone demethylation and 5-methyl-cytosine hydroxylation, thus altering global histone- and DNA-methylation, resulting in widespread alterations of epigenetic control of gene expression (90). Prolyl hydroxylases are also inhibited by D-2-hydroxyglutarate, thus leading to HIF-1α stabilization even in normoxia (91). Enhanced NADPH consumption by the reverse IDH2 reaction thus provides a selective advantage to cancer cells by promoting oncogenic epigenetic alterations and HIF activation.

In apparent contrast with this model, a recent study reported NADPH production in mitochondria of cancer cells through reductive carboxylation in the cytosol (92). In this study anchorage-independent growth of lung cancer cells was dependent upon reverse reductive carboxylation that proceeds from citrate synthesis in the cytosol *via* IDH1, its uptake in mitochondria, followed by conversion to isocitrate which is then oxidized by IDH2, with NADPH production (Figure 2). Since IDH2 operates in its oxidative decarboxylation mode, lipogenesis cannot be sustained by glutaminolysis and the citrate necessary to promote fatty acid biosynthesis reactions in the cytosol must be derived from glucose oxidation through the TCA cycle. It remains to be determined whether cytosolic reductive carboxylation is a common feature of cancer metabolism which allows the cell to sustain anabolic reactions while preserving NADPH levels in mitochondria.

Given its important role in tumorigenesis, IDH2 is being investigated as a druggable target. Enasidenib (AG-221) has been successfully tested as an inhibitor of neomorphic IDH2 mutants in hematologic malignancies (93). As AG-221 specifically inhibits the production of D-2-hydroxyglutarate by mutant IDH2, it is not effective in decreasing NADPH levels in mitochondria. In fact, inhibition of wild-type IDH2 would be advantageous in some cancers, such as melanoma, which rely on IDH2-mediated oxidative decarboxylation to maintain proper redox balance in mitochondria. In these cancers, IDH2 inhibition would induce mitochondrial oxidative stress and, probably, priming to proapoptotic stimuli.

## ME2/3

Malic enzyme catalyzes the decarboxylation of malate to pyruvate (Figure 2). Three ME isoforms are encoded by different genes. NADPH can be generated in mitochondria by the NADP-dependent ME3 isoform (less expressed) or by the ME2 isoform (more abundant), which, however, has a preference for NAD^+^. ME2 knockdown in melanoma cells increases ROS levels and impairs anchorage-independent growth *in vitro* and tumorigenicity *in vivo* (94). This is accompanied by a reduction in ATP levels, which is not surprising considering that ME2 activity leads to NADH generation. The decrease in NADH in mitochondria following ME2 silencing is exacerbated by the inhibition of the malate-aspartate shuttle resulting from the accumulation of malate. However, ROS levels are expected to drop following a decrease in the NADH/NAD^+^ ratio, which is the main factor determining the rate of superoxide formation by complex I. Thus, the increased ROS levels observed by Chang et al. (94) are probably linked to a different event, possibly a decrease in NADPH. The antitumor activity of ME2 ablation is also a consequence of AMPK activation by the increased ADP/ATP and NADP^+^/NADPH ratios. It is noteworthy that, compared to normal melanocytes, melanoma cells exhibit increased expression of ME2 and reduced expression of NADP-dependent ME3. The decreased NADPH levels observed in melanoma cells may thus result from reduced NNT activity rather than from reduced NADPH production by ME2. In the K562 erythroleukemia cell line, ME2 knockdown results in erythroid differentiation and impaired proliferation and growth in nude mice, along with reduced ATP levels and increased NAD^+^/NADH and NADP^+^/NADPH ratios (95). K562 cells also exhibited increased MitoSOX Red fluorescence following ME2 knockdown; however, this finding is not indicative of increased mtROS generation, as suggested by the authors, but only denotes increased mtROS accumulation, which is likely to result from reduced scavenging by antioxidant systems due to a decrease in NADPH levels. Again, the latter could be simply due to reduced NNT activity, secondary to decreased NADH levels. ME2 knockdown in non-small cell lung cancer cells (NSCLC) is also associated with decreased ATP levels, a drop in NADPH levels, and increased ROS levels as well as enhanced sensitivity to cisplatin treatment (96). ME2 knockdown is also predicted to have a strong impact on central carbon metabolism blocking the utilization of glucose for the synthesis of phosphatidylcholines, thus contributing to the inhibition of cell proliferation *in vitro* and tumor formation *in vivo*. Also in this case, there is no evidence that decreased NADPH levels are direct consequences of ME2 knockdown, as opposed to reduced NADH levels and decreased NNT activity.

These issues may be resolved using quantitative flux analysis, a technique that allows measurements of the fractional contribution of different pathways to the production of a metabolite by using radioisotope tracers. Using this technique, Fan et al. quantified the contribution of ME to the production of NADPH by administering [2,3,3,4,4-^2^H]glutamine and uniformly labeled glutamine ([U-^13^C]glutamine) to HEK293T embryonic kidney cells, MDA-MB-468 breast cancer cells, and baby mouse kidney epithelial cells (iBMK) (97). Deuterium-labeled glutamine allows direct measurement of hydrogen incorporation into NADPH. However, passage of deuterium from glutamine to NADPH does not exclude the contribution of the NNT. In fact, ME2 activity could result in deuterium incorporation into NADH, which subsequently donates the deuterium to NADP^+^ in the transhydrogenation reaction. However, by combining deuterium-labeling of glutamine with radioactive carbon-labeling, the direct contribution of ME to NADPH generation was estimated to equal the contribution of the PPP. Fan et al. did not perform their measurements on cell fractions, so their findings refer to NADPH production from both cytosolic ME1 and mitochondrial ME2 and ME3. However, given the results of ME2 knockdown experiments, it is reasonable to speculate that ME2 and ME3 contribute substantially to maintain the mitochondrial NADPH pool. Consequently, in mitochondria, a large fraction of NADPH is produced by ME both directly and indirectly, through the activity of the NNT. Embonic acid has recently been described as a non-competitive specific inhibitor of ME2 that reduced growth and induced senescence in the NSCLC cell line H1299 (98).

Specific inhibition of mitochondrial ME could be a promising strategy to efficiently reduce the mitochondrial NADPH pool in cancer cells, thus inducing sensitivity to proapoptotic stimuli.

## Mitochondrial Folate Cycle and Serine Pathway

**Figure 4:**
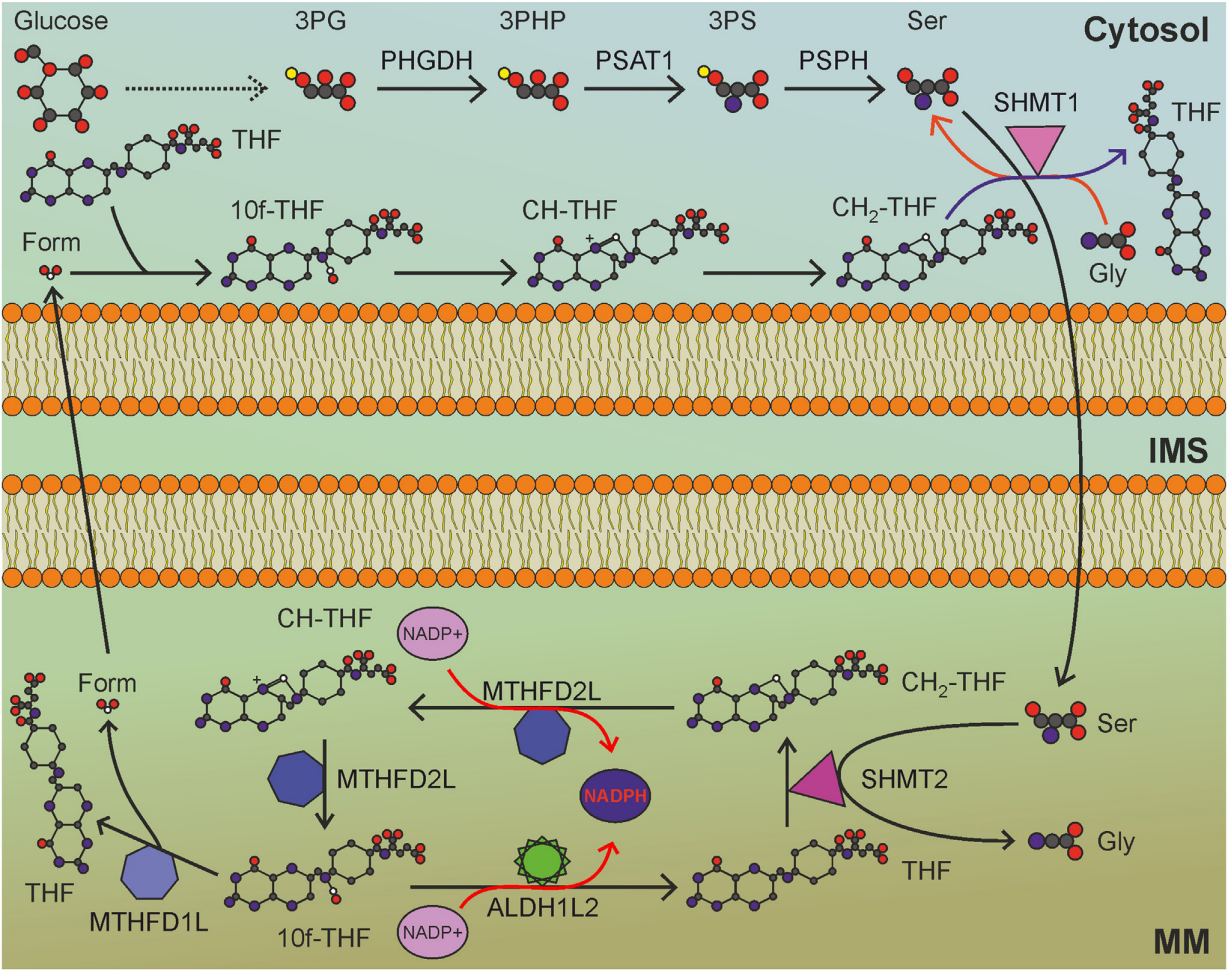
Mitochondrial folate cycle importantly contributes to NADPH production in mitochondria, and sustains anabolic reactions in the cytosol. MM, mitochondrial matrix; IMS, intermembrane space; 3PG, 3-phosphoglycerate; 3PHP, 3-phosphohydroxypyruvate; 3PS, 3-phosphoserine; Ser, serine; Gly, glycine; THF, tetrahydrofolate; CH_2_-THF, 5,10-methylene-THF; CH-THF, 5,10-methenyl-THF; 10f-THF, 10-formyl-THF; Form, formate. Gray circles indicate carbon atoms, red circles indicate oxygen atoms, blue circles indicate nitrogen atoms, and yellow circles indicate phosphate groups. Hydrogen atoms are omitted. One-carbon unit carried by THF derivatives is white-colored. Usage of THF derivatives in cytosolic anabolic reactions is not shown. Red arrows indicate reactions involved in NADPH production.

The folate cycle is one of the principal biological pathways involved in nucleotide synthesis, methionine metabolism, and methylation reactions. Long considered an NADPH-consuming pathway, the folate cycle has recently been appreciated as an important source of NADPH (97), especially in mitochondria. Indeed, the folate pathway is a complex set of reactions that transfer one-carbon units, some of which consume and some others produce NADPH. Folic acid, or vitamin B_9_, is essential to dividing cells. As human cells are not able to produce folate, it is required from the diet. The enzyme DHFR converts folate to dihydrofolate and, then, to tetrahydrofolate (THF), using NADPH as cofactor. THF is a one-carbon shuttle that exists in several derivatives characterized by different oxidation states of the shuttled carbon atom (Figure 4). The conjugation of THF with a formaldehyde group derived by the catabolism of amino acids generates 5,10-methylene-THF (CH_2_-THF), a THF derivative with an intermediate oxidation state. CH_2_-THF can be reduced to 5-methyl-THF (CH_3_-THF), with NADPH consumption, oxidized to 5,10-methenyl-THF (CH-THF), generating NADPH or used in thymidylate synthesis, regenerating THF. CH_3_-THF, along with vitamin B_12_, acts as a methyl donor in the re-methylation cycle, regenerating THF, while CH-THF is further oxidized to 10-formyl-THF (10f-THF), which is used to obtain formate or in purine synthesis. Overall, NADPH-consuming reactions outweigh NADPH-producing reactions, especially if the folate cycle is used to provide one-carbon units for re-methylation reactions. For these reasons, the folate cycle is usually considered an NADPH-consuming pathway. Recent findings, however, have changed this concept. Although one-carbon units can be provided by glycine, betaine, sarcosine, histidine, choline, and tryptophan (99), serine represents the main source of one-carbon units used to convert THF to CH_2_-THF. Interestingly, the folate cycle is compartmentalized to the cytosol and mitochondria, but most of the one-carbon units are derived by serine catabolized in mitochondria (100). The mitochondrial enzyme serine hydroxymethyltransferase 2 (SHMT2) catalyzes the generation of glycine and CH_2_-THF, which is subsequently converted by mitochondrial methylenetetrahydrofolate dehydrogenase 2 (MTHFD2) to CH-THF and, then, to 10f-THF. A small amount of the latter is used to formylate the mitochondrial initiator tRNA, but the great majority is converted by MTHFD1L to formate, which is exported to provide the cytosol with the one-carbon units necessary for purine and thymidylate synthesis. The importance of the mitochondrial folate pathway is corroborated by the observation that purinosomes—complexes of purine synthesis enzymes—colocalize with mitochondria depending on mTOR activity (101). The proposed directionality and compartmentalization of the folate cycle implies that no NADPH-consuming reaction is carried out in mitochondria, while anabolic reactions that require NADPH are compartmentalized in the cytosol.

Due to its involvement in nucleotide synthesis, the folate cycle is frequently upregulated in cancer, a property that led to the development of the antifolates aminopterin and methotrexate as chemotherapeutic agents. The ability to cope with oxidative stress is very important for cancer cells to survive proliferative stress, and dysregulation of the folate cycle could be advantageous to this end. However, MTHFD2 enzyme uses only NAD^+^ as a cofactor, thus affecting ETC activity rather than redox homeostasis. The mitochondrial NADP-dependent enzyme MTHFD2L can catalyze the conversion of CH_2_-THF to CH-THF with NADPH production, but this protein is underexpressed in cancer cells compared to normal cells (102). Another mitochondrial enzyme that can produce NADPH is 10f-THF dehydrogenase 2 (ALDH1L2), which releases CO_2_ from 10f-THF, thus wasting one-carbon units. Interestingly, ALDH1L2 is overexpressed in several cancer cell lines (103), highlighting the importance of mitochondrial NADPH pool maintenance in cancer cells.

As mentioned above, the conversion of folate to THF requires two NADPH molecules. Nevertheless, cells can directly obtain THF from the diet (an important source of THF is the intestinal microbioma), thus sparing NADPH consumption. In addition, cancer cells are characterized by opportunistic ways of nutrient acquisition (70), including entosis—the digestion of entire living cells—with acquisition of pre-formed THF.

Serine is the principal source of one-carbon units that feed the folate cycle. Thus, it is not surprising that serine uptake and *de novo* serine synthesis are commonly upregulated in cancer, along with genes of the mitochondrial folate pathway. Nuclear factor (erythroid-derived 2)-like 2 (NRF2), the master regulator of antioxidant responses, promotes transcription of mitochondrial folate cycle-related genes through interacting with ATF4 (104), whose transcription is activated by mTOR (105), thus integrating the antioxidant response with energy-sensing pathways. The involvement of NRF2 in upregulation of the mitochondrial folate cycle in cancer highlights the important role that this pathway plays in the antioxidant response. Serine catabolism in mitochondria produces glycine, essential for glutathione and purine synthesis, and contributes to mitochondrial redox balance in cancer cells exposed to hypoxia (106). Ye et al. showed that SHMT2 is upregulated in Myc-amplified neuroblastoma and glioblastoma cell lines upon exposure to hypoxia. Accordingly, SHMT2 knockdown results in decreased NADPH levels, increased mtROS, reduced GSH levels, hypoxia-triggered cell death, and impaired tumor growth *in vivo*. The upregulation of SHMT2 in cancer correlates with the increased expression of phosphoglycerate dehydrogenase (PHGDH), the enzyme that catalyzes the first, limiting, step in the *de novo* serine synthesis pathway. Serine is synthesized from the glycolytic intermediate 3-phosphoglycerate (3PG), which is oxidized by PHGDH to 3-phosphohydroxypyruvate, subsequently used by phosphoserine aminotransferase 1 (PSAT1) in a transamination reaction with glutamate, leading to 3-phosphoserine (3PS). Finally, 3PS is converted to serine by phosphoserine phosphatase (PSPH). Enhanced glycolysis in cancer cells has a very important role in antioxidant defenses, providing glucose-6-phosphate to the PPP in the cytosol and 3PG to serine synthesis, and the folate cycle in mitochondria.

Hypoxia upregulates PHGDH, PSAT1, PSPH, SHMT2, MTHFD2, and MTHFD1L, thus protecting breast cancer stem cells from oxidative damage (107). Consistent with this notion, PHGDH knockdown abrogates breast cancer stem cell survival under hypoxia, without impairing cancer cell proliferation, and increases oxygen consumption rate and extracellular acidification rate as a consequence of reduced glucose shunting to the serine synthesis pathway. PHGDH-ablated breast cancer cells are sensitized to ROS-inducing chemotherapy and, despite being tumorigenic, do not generate lung metastases *in vivo*, due to loss of the cancer stem cell population. Thus, PHGDH overexpression and consequent increased NADPH production from the mitochondrial folate cycle are important mechanisms determining cancer stem cell maintenance under oxidative stress.

Glycine contributes to NADPH production through the Serine—One carbon—Glycine (SOG) pathway, which is upregulated in several cancers and is associated with expression of Myc target genes (108). Through the serine synthesis pathway, cytosolic and mitochondrial one-carbon cycles with nucleotide synthesis reactions, and the glycine cleavage system, the SOG pathway supplies the ATP, NADPH, and purines needed by highly proliferating cancer cells to sustain biosynthetic pathways and to cope with oxidative stress in Myc-driven cancers.

Dietary serine is transported inside cells by the SLC7A10 transporter, while *de novo* serine synthesis is activated in normal cells during fasting. On the contrary, PHGDH-overexpressing cancer cells are addicted to *de novo* serine synthesis, whose inhibition leads to the blockade of exogenous serine incorporation into purines (109). This paradoxical effect might be due to the fact that, in the absence of serine synthesis from PHGDH, SHMT1 catalyzes the futile generation of serine from glycine, thus depleting cytosolic CH_2_-THF. It is also possible that PHGDH inhibition in serine synthesis-addicted cancer cells shifts the equilibrium of the SHMT2 reaction toward serine, thus inverting the mitochondrial folate cycle and blocking NADPH production in mitochondria. This raises the possibility to target the antioxidant response in cancer cell mitochondria by inhibiting the serine synthesis pathway and folate cycle. The inhibition of SHMT enzymes could be achieved using antifolate drugs (110, 111), antimetabolites that are already used in the clinic. It is noteworthy that, besides targeting DHFR, methotrexate inhibits many biochemical reactions involving NAD and NADP as cofactors, leading to a broad inhibition of NADPH production in cancer cells [reviewed in Ref. (112)]. However, all antifolate drugs are more specific for cytosolic SHMT1 than for mitochondrial SHMT2 and their anticancer effect is principally mediated by cytosolic purine depletion. SHMT2-specific inhibitors, whose action would lead to NADPH depletion in mitochondria, are not currently available. The second promising approach to lower the mitochondrial NADPH pool is through inhibition of the serine synthesis pathway. PHGDH inhibition in highly proliferating cancer cells addicted to *de novo* serine synthesis has the potential to deplete one-carbon units in the mitochondrial folate cycle, thus lowering the mitochondrial NADPH pool and inducing oxidative stress. This approach might be much less effective in cancer cells with low PHGDH, suggesting PHGDH expression as a predictive marker of response to therapies based on inhibitors of serine synthesis.

Inhibition of the folate cycle and serine synthesis pathway would impair the principal route through which the NADPH pool is maintained in mitochondria of cancer cells, thus sensitizing them to ROS-induced cell death.

## Concluding Remarks

Reactive oxygen species act as a double-edged sword in cancer cells. On one hand, increased mtROS production drives sustained mitogenic signaling, oncogenic transformation, genomic instability, and cell cycle checkpoint evasion—all hallmarks of cancer. On the other hand, excessive accumulation of H_2_O_2_ leads to irreversible protein modification, oxidative damage to lipids and nucleic acids, proliferative signaling blockade, and, ultimately, cell death. This is particularly important in mitochondria, which harbor several proapoptotic proteins. Apoptotic cell death is triggered by the release of cytochrome *c* from mitochondria through the proapoptotic proteins BAX and BAK. Excessive ROS accumulation in mitochondria can also drive release of cytochrome *c* through the activation of the permeability transition pore (55, 56) or through BAX/BAK gateway opening (57). In addition, mtROS might also increase the mobilization of cytochrome *c* from the cristae compartments through the proteolytic processing of optic atrophy 1, a dynamin-related protein that is involved in mitochondrial fusion and cristae remodeling (113, 114).

**Figure 5:**
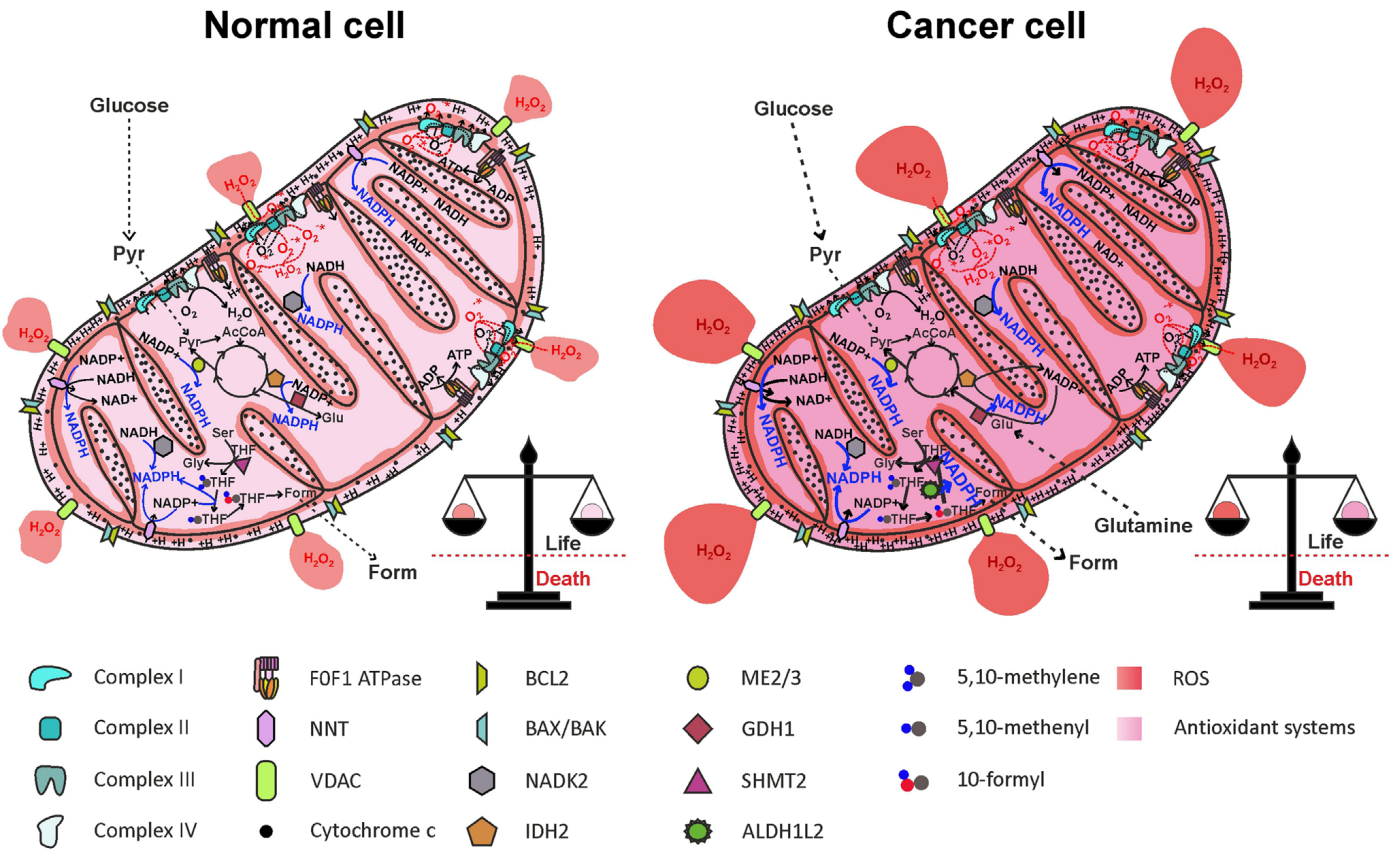
Reactive oxygen species (ROS) generation by the electron transport chain (ETC) and NADPH production in mitochondria. One-electron reduction of oxygen at the level of complexes I, II, and III of the ETC generates $O_{2}^{-•}$, rapidly dismutated to H_2_O_2_, which diffuses in the mitochondrial matrix and in the cytosol, through the VDAC. ROS are counterbalanced by NADPH generation through NNT, NADK2, IDH2, ME2/3, GDH1, and the folate cycle, maintaining redox homeostasis. In cancer cell, NADPH production pathways are more active, to offset increased ROS levels generated by oncogenic pathways. AcCoA, acetyl-CoA; ALDH1L2, 10-formyl-THF dehydrogenase 2; Form, formate; GDH1, glutamate dehydrogenase 1; Glu, glutamate; Gly, glycine; IDH2, isocitrate dehydrogenase 2; ME2/3, malic enzyme 2/3; NADK2, mitochondrial NAD kinase; NNT, nicotinamide nucleotide transhydrogenase; Pyr, pyruvate; Ser, serine; SHMT2, serine hydroxymethyltransferase 2; THF, tetrahydrofolate; VDACs, voltage-dependent anion channels.

Increased ROS levels may represent an Achilles’ heel of cancer cells that may be exploited therapeutically, as a small increase in ROS levels may overcome the toxic threshold, leading to mitochondrial cristae remodeling and apoptotic cell death. High ROS levels in cancer cells, however, are counterbalanced by increased antioxidant defenses (Figure 5). NADPH production in cancer cell mitochondria is critical to maintain glutathione and other scavenging molecules in a reduced state, preventing oxidative damage to mitochondrial structures and apoptosis. In this review, we analyzed the different mitochondrial NADPH-producing mechanisms, pointing out to the folate cycle and ME (partly through the activity of the NNT) as the principal contributors to mitochondrial NADPH pool maintenance in cancer cells mitochondria. The role of IDH2 and GDH1 appears to be cancer type-dependent and contributes to NADPH accumulation in mitochondria only in the context of glutamine anaplerosis of forward TCA cycle.

**Figure 6:**
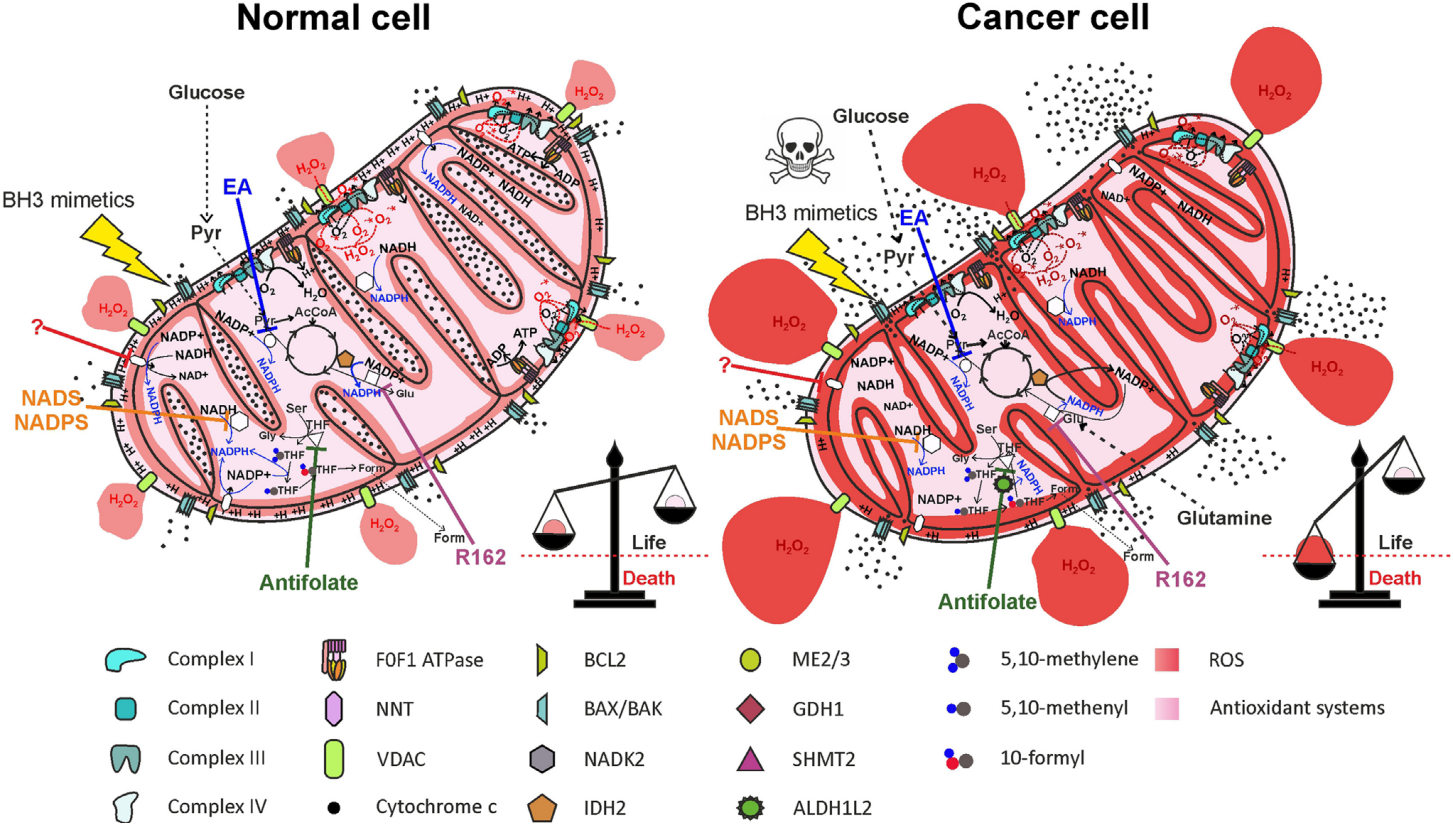
Therapeutic targeting of NADPH producing pathways in mitochondria impairs antioxidant defenses, leading to selective elimination of cancer cells. Inhibition of nicotinamide nucleotide transhydrogenase (NNT) (by a yet unidentified drug), of mitochondrial NAD kinase (NADK2) by thionicotinamide adenine dinucleotide (NADS) or by thionicotinamide adenine dinucleotide phosphate (NADPS), of ME2/3 by embonic acid (EA), of serine hydroxymethyltransferase 2 (SHMT2) by antifolate drugs, or of glutamate dehydrogenase 1 (GDH1) by R162 impairs NADPH production both in normal cells and in cancer cells. Elevation of reactive oxygen species (ROS) levels in cancer cells above the toxic threshold induces mitochondrial depolarization and release of cytochrome *c* in the intermembrane space (IMS). Treatment of cells with BH3 mimetics (such as ABT-199 or ABT-263), which cause opening of the BAX/BAK gateway, leads to massive release of cytochrome *c* in the cytosol and selective cell death of cancer cells. Inhibited proteins are white-colored.

A thorough study by Fan and colleagues (97) provided evidence that ~40% of the NADPH pool is derived by folate cycle and ~30% is derived by ME in HEK293T embryonic kidney cells, MDA-MB-468 breast cancer cells, and iBMK. Moreover, NNT is estimated to generate about half of the mitochondrial NADPH pool in healthy cells (60), reinforcing the notion of a cooperation between NNT and ME in NADPH production, as suggested above. In HEK293T transformed cells, however, NNT knockdown did not significantly change the NADPH/NADP^+^ ratio (97). The folate cycle is strongly upregulated in cancer cells due to its involvement in cell proliferation. Given the importance of mitochondrial folate cycle to provide the cytosol with formate, necessary to sustain purine and thymidylate synthesis through the cytosolic folate cycle, a large proportion of NADPH in mitochondria of cancer cells is derived by oxidation of THF derivatives. In particular, ALDH1L2 overexpression in cancer cells importantly contributes to NADPH generation. In breast cancer cells, PHGDH knockdown and consequent impairment of the mitochondrial folate cycle led to a decrease in NADPH level of ~50 and ~30% in MDA-MB-231 and in MCF7 cell lines, respectively (107), thus confirming the prediction by Fan and colleagues. Interestingly, exposure of breast cancer cell lines to hypoxia led to a repressed expression of G6PD, while increasing the expression of genes involved in serine synthesis pathway and mitochondrial folate cycle (107). These data suggest that HIF-1α is involved in the switch from cytosolic to mitochondrial NADPH production by upregulating the mitochondrial folate cycle. Probably, NADPH production by the mitochondrial folate cycle is particularly important during hypoxia, a condition frequently found in solid tumors and associated with resistance to radiotherapy and chemotherapy [reviewed in Ref. (115)]. The importance of ME in mitochondrial NADPH production has been recently highlighted in pancreatic cancer. Genomic deletion of *ME2* led to a dramatic drop in NADPH levels in pancreatic cancer cell lines exposed to doxorubicin-induced oxidative stress (116). As in the case of the mitochondrial folate cycle, the role of ME in mitochondrial NADPH production is especially important in hypoxic conditions (117). Knockdown of ME2 in the NSCLC cell line A549 led to a decrease of about two-thirds in NADPH/NADP^+^ ratio (96). The contribution of NNT to mitochondrial redox homeostasis in cancer cells is not simply evaluable due to the cooperation with ME; however, hypoxia induces an increased mitochondrial NADH/NAD^+^ ratio, which drives NADPH production by the NNT, thus enhancing scavenging of mtROS produced by the ETC (117). Overall, cancer cells resist hypoxia by driving mitochondrial NADPH production through mitochondrial folate cycle, ME, and NNT. Inhibition of mitochondrial NADPH generation by targeting each of the described pathways impairs antioxidant defenses both in normal and in cancer cells, but only in the latter ROS accumulation is likely to exceed the toxic threshold, leading to massive release of cytochrome *c* and cell death (Figure 6).

Importantly, despite being compartmentalized inside cells, cytosolic and mitochondrial NADPH pools interact with each other. In fact, as described above, IDH2 can catalyze the reductive carboxylation of α-ketoglutarate to isocitrate, using NADPH. Isocitrate can then be converted to citrate, which is exported to the cytosol, being reconverted to isocitrate. The cytosolic enzyme IDH1, finally, catalyzes the oxidative decarboxylation of isocitrate to α-ketoglutarate, producing NADPH (118). This mitochondrial NADPH shuttle system implies that NADPH-producing pathways in mitochondria could partially contribute to cytosolic NADPH pool maintenance, mostly in cancer cells using IDH2 to sustain reductive carboxylation. Inhibition of mitochondrial NADPH production, consequently, could affect cytosolic NADPH pool.

The identification of novel drugs targeting the mechanisms by which cancer cells maintain their redox balance and avoid cell death may pave the way to new combinatorial anticancer therapies that exploit the concept of mitochondrial priming to overcome apoptosis resistance to chemotherapeutics currently used in the clinic.

## Author Contributions

FC wrote the manuscript and created the figures. VC wrote the manuscript.

## Conflict of Interest Statement

The authors declare that the research was conducted in the absence of any commercial or financial relationships that could be construed as a potential conflict of interest.
